# Phase I Study of mTORC1/2 Inhibitor Sapanisertib (CB-228/TAK-228) in Combination with Metformin in Patients with *mTOR/AKT/PI3K* Pathway Alterations and Advanced Solid Malignancies

**DOI:** 10.1158/2767-9764.CRC-22-0260

**Published:** 2024-02-12

**Authors:** Vivek Subbiah, Niamh Coleman, Sarina A. Piha-Paul, Apostolia M. Tsimberidou, Filip Janku, Jordi Rodon, Shubham Pant, Ecaterina E. Ileana Dumbrava, Siqing Fu, David S. Hong, Shizhen Zhang, Ming Sun, Yunfang Jiang, Jason Roszik, Juhee Song, Ying Yuan, Funda Meric-Bernstam, Aung Naing

**Affiliations:** 1 Department of Investigational Cancer Therapeutics, The University of Texas MD Anderson Cancer Center, Houston, Texas.; 2 Department of Biostatistics, MD Anderson Cancer Center, Houston, Texas.; 3 Khalifa Institute for Personalized Cancer Therapy, MD Anderson Cancer Center, Houston, Texas.; 4 Department of Surgical Oncology, MD Anderson Cancer Center, Houston, Texas.

## Abstract

**Background:**

Sapanisertib (CB-228/TAK-228) is a potent, selective ATP-competitive, dual inhibitor of mTORC1/2. Metformin is thought to inhibit the mTOR pathway through upstream activation of 5′-AMP-activated protein kinase (AMPK) suggesting combination therapy may enhance antitumor activity of sapanisertib. We report preliminary safety, tolerability, and efficacy from the dose-escalation study of sapanisertib in combination with metformin in patients with advanced solid tumors.

**Methods:**

Patients with advanced metastatic solid tumors resistant or refractory to standard treatment, with and without *mTOR/AKT/PI3K* pathway alterations, received sapanisertib 3 or 4 mg daily together with metformin once to three times daily (500–1,500 mg). All patients underwent 14-day titration period for metformin in cycle 1. Tumor measurements were performed following cycle 2 and subsequently every 8 weeks.

**Results:**

A total of 30 patients were enrolled across four cohorts (3 mg/500 mg; 3 mg/1,000 mg, 4 mg/1,000 mg; 4 mg/1,500 mg). 19 were female (63%), median age was 57 (range: 30–77), all were Eastern Cooperative Oncology Group performance status 1. Tumor types included sarcoma (6), breast (4), ovarian (4), head and neck (3), colorectal (2), lung (2), renal cell (2), endometrial (2), gastroesophageal junction (1), prostate (1), stomach (1), urachus (1), and cervical cancer (1). Median number of prior lines of therapy was 4. Most common genomic alterations included *PIK3CA* (27%), *PTEN* (17%), *AKT1/2* (10%), *mTOR* (10%). Of 30 patients evaluable for response, 4 patients achieved partial response (PR); 15 patients achieved stable disease (SD) as best response. Disease control rate (PR+SD) was 63%. Of the responders in PR, 3 of 4 patients had documented *PTEN* mutations (3/5 patients enrolled with *PTEN* mutations had PR); 2 of 4 of patients in PR had comutations (patient with leiomyosarcoma had both *PTEN* and *TSC;* patient with breast cancer had both *PTEN* and *STK11*); 1 of 4 patients in PR had *AKT* and *mTOR* mutation; tumor types included leiomyosarcoma (*n* = 2), breast (*n* = 1), and endometrial cancer (*n* = 1). Most common treatment-emergent adverse events included nausea, anorexia, diarrhea, and rash. Grade (G) 3–5 treatment-related adverse events included hyperglycemia (4/30; 13%), fatigue (2/30; 7%), hypertriglyceridemia (1/30; 3%), rash (2/20; 7%), diarrhea (2/30; 7%), creatinine increase (1/30; 3%), acidosis (1/30; 3%). No dose-limiting toxicities (DLT) were reported in the 3 mg/500 mg cohort. One of 6 patient had DLT in the 3 mg/1,000 mg cohort (G3 diarrhea) and 2 of 11 patients had DLTs in the 4 mg/1,500 mg cohort (G3 fatigue, G3 rash). 4 mg/1,000 mg was defined as the MTD.

**Conclusions:**

The safety profile of mTORC1/2 inhibitor sapanisertib in combination with metformin was generally tolerable, with antitumor activity observed in patients with advanced malignancies harboring *PTEN* mutations and *AKT/mTOR* pathway alterations.

**Significance:**

Sapanisertib (CB-228/TAK-228) is a potent, selective ATP-competitive, next-generation dual inhibitor of mTORC1/2. Metformin is thought to inhibit the mTOR pathway through upstream activation of AMPK suggesting combination therapy may enhance antitumor activity of sapanisertib. This dose-escalation study of sapanisertib and metformin in advanced solid tumors and mTOR/AKT/PI3K pathway alterations, demonstrates safety, tolerability, and early clinical activity in advanced malignancies harboring PTEN mutations and AKT/mTOR pathway alterations.

Clinical trial information: NCT03017833

## Introduction

The PI3K/v-Akt Murine Thymoma Viral Oncogene (AKT)/mTOR pathway plays a diverse role in regulation of several cellular functions such as cellular growth, proliferation, and survival (1). This pathway is frequently dysregulated in various cancers, such endometrial, cervical, lung, prostate, skin, and breast cancer (2–4), and mTOR is a key node and central regulator in this pathway.

mTOR exists in two physically and functionally distinct protein signaling complexes, one with raptor, which is sensitive to the mTOR inhibitor rapamycin, and the other with rictor, which is rapamycin insensitive (5). The first, mTOR complex 1 (mTORC1), phosphorylates 4EBP1 (the eukaryotic translation initiation factor 4E-binding protein 1) and p70S6 kinase and results in translation of proteins involved in cell-cycle progression. The second, mTOR complex 2 (mTORC2), has been shown to directly phosphorylate and activate the upstream kinase AKT at serine 473, which promotes proliferation and survival pathways (6). The rapalogs, such as everolimus, exert their inhibitory action predominantly via mTORC1, while their inhibitory effect on mTORC2 is limited or weak (7–10). Consequently, continued signaling through significant pathway feedback loops, results in upregulation of AKT, which has the undesirable effect of accelerating cell proliferation, antagonizing the antiproliferative effect of mTOR inhibition (11). Thus, next-generation dual inhibitors of mTORC1 and mTORC2 have been developed, and preclinical models have supported the potency of this dual inhibition strategy (12–15).

Recently, mTOR inhibitors have been approved by the FDA for the treatment of advanced renal, breast and several other cancers (7–10, 16). Sapanisertib (CB-228, formerly TAK-228 and MLN0128) is a potent, selective ATP-competitive, dual inhibitor of mTORC1/2, regulated by upstream receptor tyrosine kinases, such as insulin-like growth factor-1 receptor (IGF-1R). In early-phase clinical trials, single-agent sapanisertib was well tolerated and demonstrated preliminary antitumor activity in renal cell carcinoma and endometrial cancer (17).

Metformin, a common antidiabetic medication has been repurposed as antineoplastic agent to enhance the effect of chemotherapy in certain cancers (18). The mechanisms attributed to antihyperglycemic effects of metformin are manifold, including activation of 5′-AMP-activated protein kinase (AMPK), decreased production of cyclic AMP, suppression of mitochondrial complex I of the electron transport chain, glycerophosphate dehydrogenase targeting, and gut microbiome alterations (19). Mechanistically, metformin inhibits the mTOR pathway through upstream activation of AMPK, which results in phosphorylation and activation of the tumor suppressor gene TSC2. This decreases AKT activation, and exerts an inhibitory effect on mTOR (20). Preclinically, metformin-induced activation of AMPK has been shown to disrupt cross-talk between insulin/IGF-1R and G protein–coupled receptors signaling in pancreatic cancer cells, (21) and inhibit cell proliferation, reduce colony formation, and inhibit MAP kinase, AKT, and mTOR in estrogen receptor–positive and –negative, as well as ERBB2-normal and -overexpressing breast cancer cell lines (22). In addition, population studies have reported a decrease in the incidence and mortality rate in patients with cancer taking metformin for management of diabetes (23–26).

Taken together, these observations suggest that combination therapy using metformin and sapanisertib which both target the mTOR pathway could complement and enhance antitumor activity of sapanisertib. Here, we report the preliminary safety, tolerability, and efficacy from the dose-escalation study of sapanisertib in combination with metformin in patients with advanced solid tumors and mTOR/AKT/PI3K pathway alterations.

## Materials and Methods

### Study Design

**FIGURE 1:**
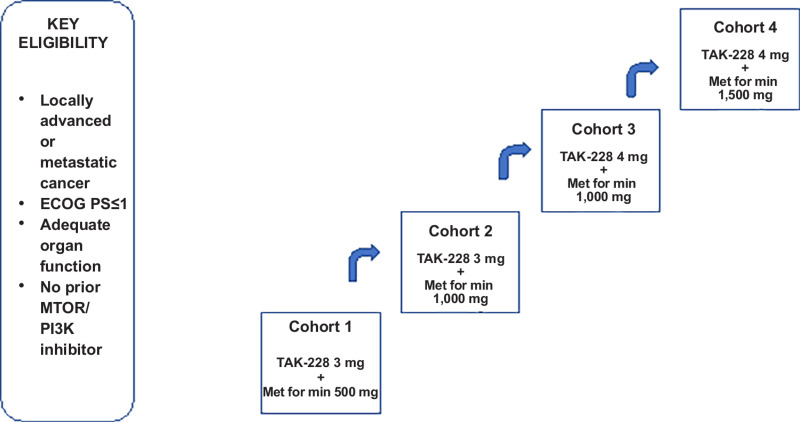
Trial schema.

This was an open-label, single-center investigator-initiated phase I clinical trial that employed a 3 + 3 dose-escalation design and was conducted at The University of Texas MD Anderson Cancer Center (MDACC; Houston, TX) per Institutional Review Board guidelines. The primary endpoint was to evaluate the safety and tolerability of sapanisertib in combination with metformin, to determine MTD and dose-limiting toxicities (DLT) of the combination in patients with advanced cancers refractory to standard therapy. The secondary objective was to evaluate preliminary antitumor efficacy of this treatment per RECIST version 1.1 (RECIST v1.1; ref. 27). Dose escalation included four dose levels. During dose escalation, patients received one of four dosing schedules: sapanisertib 3 or 4 mg once daily in combination with metformin one to three times daily (500 mg/1,000 mg/1,000 mg/1,500 mg; Fig. 1). The dosing was based on data from previous phase I studies with this agent. Study INK128-001 was a phase I, open-label study of single-agent TAK-228 administered to patients diagnosed with advanced solid malignancies, including, but not limited to, colorectal, renal, endometrial, and urothelial tumors. Four dosing schedules were evaluated (once daily, every week, once daily × 3 days every week, and once daily × 5 days every week). The MTDs for the four schedules investigated in INK128-001 were determined to be 6 mg for once daily dosing, 16 mg for once daily × 3 days every week dosing, 10 mg for once daily × 5 days every week dosing, and 40 mg for every week dosing. On the basis of these data and pharmacokinetic data, it was decided to use the Q Day dosing schema in combination with metformin.

All patients underwent 14-day titration period for metformin in cycle 1 to limit side effects and commenced sapanisertib on cycle 1 day 15; thereafter, treatment was administered in 4-week cycles. Tumor measurements were performed following cycle 2 and subsequently every 8 weeks. Data cut-off date was March 31, 2021.

DLT was defined if the events occurred within the days 15–42 of the first cycle. Definitions for DLTs, as predetermined in the study protocol, were: any grade ≥3 non-hematologic toxicity, except for the following: grade 3 hyperglycemia lasting ≤14 days (all patients should have received optimal antiglycemic treatment, including insulin, as clinically indicated); grade 3 rash lasting ≤3 days (all patients should have received topical steroid treatment, oral antihistamines, and oral steroids, if indicated); inadequately treated grade 3 nausea and/or vomiting and grade 3 diarrhea (all patients should have received optimal antiemetic and/or antidiarrheal prophylaxis and/or treatment as indicated); grade 4 neutropenia lasting >7 days in the absence of growth factor support; any grade neutropenia of any duration accompanied by fever ≥38.5°C and/or systemic infection; any other ≥ grade 4 hematologic toxicity; inability to administer at least 75% of planned doses of TAK-228 within cycle 1, due to study drug-related toxicity; grade 3 thrombocytopenia with bleeding; any death not clearly due to underlying disease or extraneous causes.

### Patients

The study accrual period was from March 12, 2018 to 2020. Eligible patients included patients with metastatic or advanced solid tumors not amendable to standard therapy, an Eastern Cooperative Oncology Group (ECOG) performance status (PS) 0–1 and adequate hematologic, hepatic, and renal function. Patients had to have the ability to swallow oral medications. Diabetic patients with well-controlled diabetes (i.e., normal range HbA1C ≤7%) were permitted and allowed to be on antidiabetic treatment other than metformin. Notable exclusion criteria included poorly controlled diabetes mellitus [defined as glycosylated hemoglobin (HbA1c) >7%], prior treatment with dual PI3K/mTOR inhibitors, TORC1/2 inhibitors or TORC1 inhibitors, significant cardiovascular or pulmonary disease, intercurrent uncontrolled illness, patients receiving corticosteroids (either intravenous or oral steroids, excluding inhalers or low-dose hormone replacement therapy) within 1 week before administration of the first dose of study drug, and malabsorption due to prior gastrointestinal (GI) surgery, GI disease (e.g., patients with enteric stomata were excluded). Patients with treated, stable brain metastases (defined as no evidence of disease progression for ≥3 months before the first dose of study drug, no hemorrhage after treatment, off-treatment with dexamethasone for 4 weeks before administration of the first dose of sapanisertib, no ongoing requirement for dexamethasone or antiepileptic drugs) were permitted.

### Assessments

Patients who received at least one dose of any study drug were included in the safety population. DLT-evaluable population was defined as all patients who either experienced DLT during cycle 1 or completed treatment with at least 75% of the planned doses of any study drug in cycle 1 and had sufficient follow-up data to allow the investigators to determine whether DLT occurred. The response-evaluable population was defined as all patients who had measurable disease according to RECIST v1.1, who had received at least one dose of any study drug, and who have at least one available postbaseline response assessment as per RECIST v1.1. Response was assessed according to the RECIST v1.1 after every two treatment cycles. Adverse events (AE) were assessed using the NCI Common Terminology Criteria for Adverse Events, version 4.03.

### Statistical Analysis

No formal hypotheses were tested, and analyses were descriptive and exploratory. Nonparametric correlations were determined with Spearman rank correlation coefficient.

### Ethical Approval and Consent to Participate

The protocol was approved by the Institutional Review Board at The University of Texas MDACC (Houston, TX). The study was conducted in accordance with the Declaration of Helsinki and the International Conference on Harmonization Good Clinical Practice guidelines. All patients provided written informed consent before enrollment.

### Data Availability Statement

The datasets used and/or analyzed during the current study are available from the corresponding author on reasonable request.

## Results

### Patients

The patients reported here include all patients with heavily pretreated advanced solid tumors treated as part of a dose-escalation study. A total of 30 patients with advanced or metastatic solid tumors received treatment with sapanisertib and metformin on a range of dose levels. Baseline demographics and characteristics are shown in Table 1. Nineteen patients were female (63%), 11 patients were men (37%), and the overall median age was 57 (range: 30–77 years). The most frequent tumor types enrolled were sarcoma (6), breast (4), ovarian (4), head and neck (3), colorectal (2), lung (2), renal cell (2), endometrial (2), gastroesophageal junction (1), prostate (1), stomach (1), bladder (1), and cervical cancer (1). Patients were enrolled across four cohorts and received sapanisertib in combination with metformin in the following dose combinations (3 mg/500 mg; 3 mg/1,000 mg, 4 mg/1,000 mg; 4 mg/1,500 mg; Fig. 1). Nineteen patients were female (63%), median age was 57 (range: 30–77), all patients were ECOG PS 1. Median number of prior lines of therapy was 4 and 70% of patients (*n* = 21) and more than three lines of therapy.

**TABLE 1:** Baseline patient demographics

Characteristic	Number (%)
Gender	
Female	19 (63)
Male	11 (37)
Median age at study enrollment, years (range)	57 (30–77)
Ethnicity	
Asian	3 (10)
White	21 (70)
Hispanic	2 (7)
African American	3 (10)
Other	1 (3)
Number of metastatic sites	
≤3	27 (90%)
>3	3 (10%)
Disease type	
Breast	4 (13)
Colon	2 (7)
Head and neck	3 (10)
Lung	2 (7)
Ovarian	4 (13)
Renal cell carcinoma (RCC)	2 (7)
Sarcoma	6 (20)
Others (endometroid 2, gastroesophageal junction 1, prostate 1, stomach 1, urachus 1, cervix 1)	7 (23)
ECOG PS	
0	0 (0)
1	30 (100)
Number of prior therapies (range)	(0–11)
1–2	9 (30)
3–4	14 (47)
5 or more	7 (23)

### DLTs and MTD Determination

Dose escalation, DLTs, and MTDs are summarized in Supplementary Table S1; dose was escalated to level 4. No DLTs were reported in the first cohort treated, dose level 1 (3 mg/500 mg). One patient (1/6) experienced DLT of grade 3 diarrhea in the second cohort, dose level 2 (sapanisertib 3 mg/ metformin 1,000 mg), which was managed supportively with no dose reductions. There were no DLTs in dose level 3. Two further patients (2/11) had DLTs in dose level 4 (sapanisertib 4 mg/metformin 1,500 mg cohort), one patient with grade 3 fatigue, which required two dose-level reductions for sapanisertib; one patient with grade 3 rash, which required reduced dose level on restarting medication. Both these patients were able to continue treatment after dose modification. Of 30 treated patients, 4 patients (13.3%) had dose changes with either drug. Three patients (10%) had dose reduction with sapanisertib. On the basis of these findings, sapanisertib 4 mg in combination with metformin 1,000 mg was defined as the MTD and recommended phase II dose.

### Treatment Response

**FIGURE 2:**
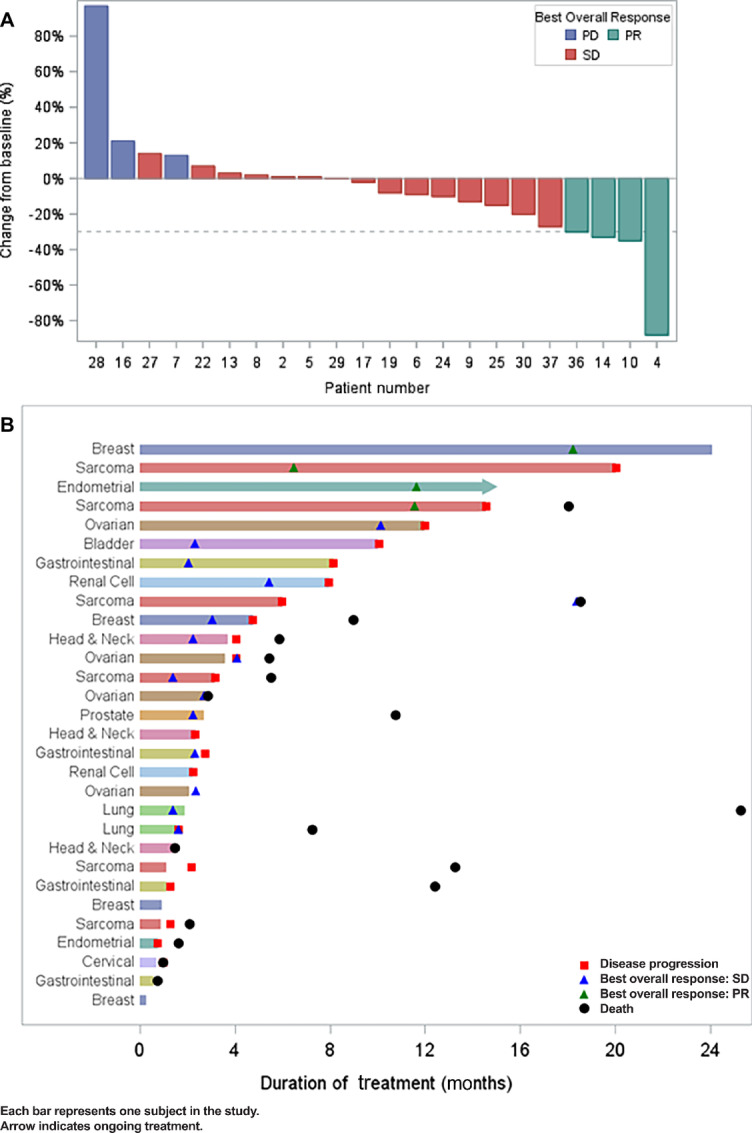
**A,** Waterfall plot showing best overall response of evaluable patients on trial. Among 30 patients in the dataset, 24 patients were evaluated for best overall response and 6 patients were not evaluated. Among 24 with best overall response, 4 had PR, 15 had SD, and 5 had PD. For these 24 patients, 22 had percent change from baseline tumor size measured. Two patients who had progression due to unequivocal progression in non-target lesions did not have a tumor size measurement. **B,** Swimmers plot. All 30 patients treated on study are included. PR, SD, PD, death, and ongoing treatment are indicated.

Overall, there were 24 response-evaluable patients per RECIST v1.1. For these 24 patients, 22 had percent change from baseline tumor size measured (Fig. 2). Two patients who had progression due to unequivocal progression in non-target lesions did not have a tumor size measurement. Tumor response across cohorts is summarized in Table 2. Of the 24 evaluable patients, 19 patients (79%) had documented disease control [defined as stable disease (SD) or partial response (PR) per RECIST v1.1 of more than 16 weeks]. Notably, 4 patients (17%) achieved PR as best tumor response; these responses were in patients with leiomyosarcoma (*n* = 2), breast cancer (*n* = 1), and endometrial cancer (*n* = 1; Fig. 2). PRs were achieved at dose level 1 (*n* = 1), dose level 2 (*n* = 1), and dose level 3 (*n* = 2). The most frequent reason for study discontinuation was progressive disease (PD) in 63% of all patients. Overall, the median overall survival was 18 months [95% confidence interval (CI), 7.2–NE] and median progression-free survival was 6.0 months (95% CI, 2.8–14.6); median follow-up was 19 months (Supplementary Fig. S1).

**TABLE 2:** Tumor response according to RECIST v1.1 (investigator assessment) in response-evaluable patients

Best confirmed response	Number of patients
Partial response (PR)	4 (13%)
Stable disease (SD)	15 (50%)
Progressive disease (PD)	5 (17%)
Not evaluable	6 (20%)

### Genomic Alterations

**FIGURE 3:**
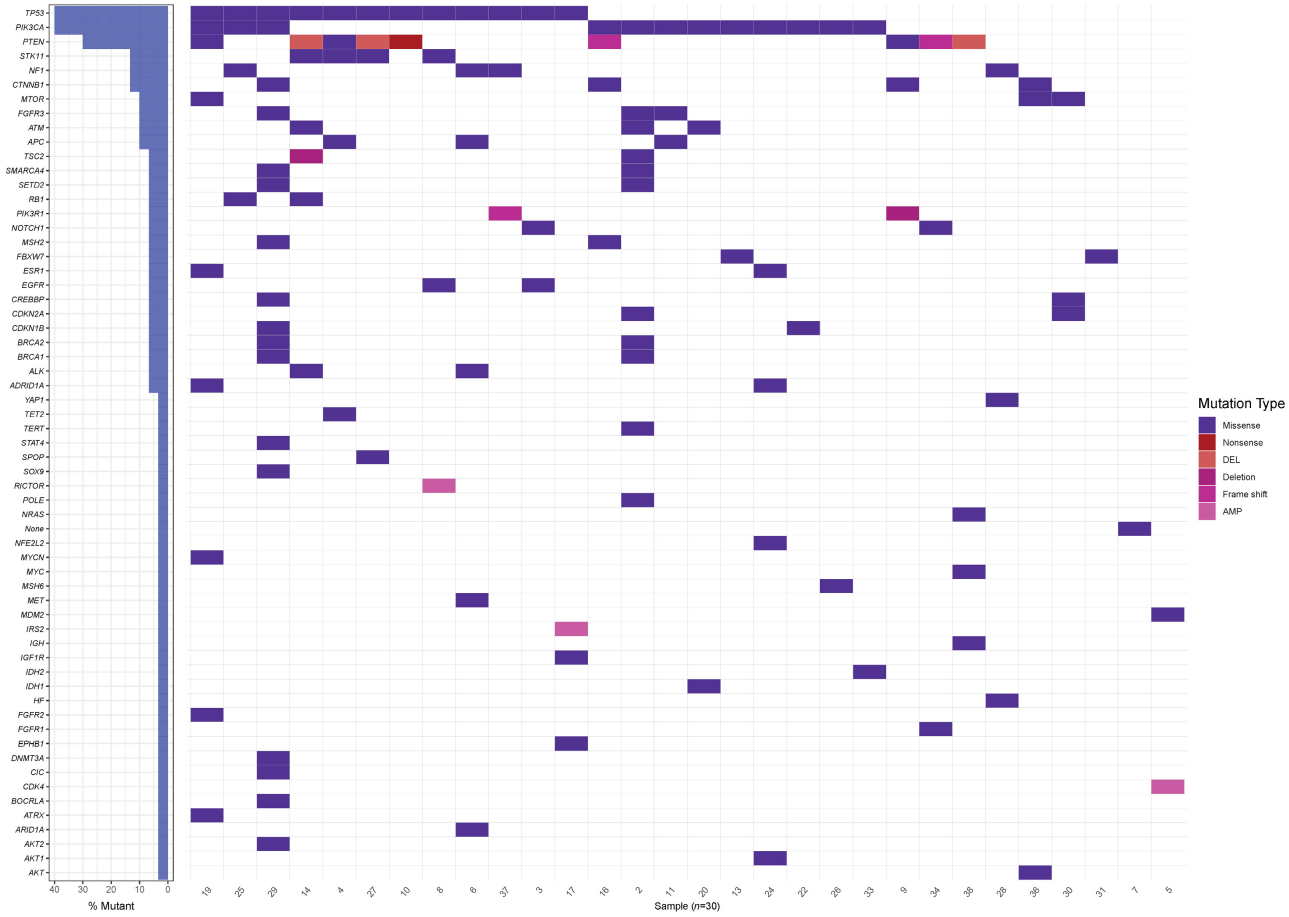
Oncoplot showing mutations and co-occurring alterations in the patients enrolled in this clinical trial.

Three out of 4 patients in PR had documented PTEN mutations (3 out of 5 patients with PTEN mutations enrolled on study achieved PR; Supplementary Table S2); 2 of these patients (2/4) had concomitant alterations in the PI3K pathway, that is, these patients had documented lesions in PTEN and STK11 (patient with breast cancer), PTEN and TSC2 (patient with leiomyosarcoma), respectively. The remaining patient in PR had dual mutations in AKT and mTOR; this patient with endometrial carcinoma (and dual AKT E17K and mTOR mutation A1459D) remains on treatment (currently on 26 months of treatment), in ongoing PR. Of all treated patients, the most common genomic alterations included mutations in PIK3CA (27%), PTEN (20%), AKT1/2 (10%), mTOR (10%; Supplementary Fig. S1; Supplementary Table S2). Comutations were frequently observed: 50% of treated patients had ≥2 genomic aberrations, including PIK3CA + NF1, AKT1, TSC2, PTEN; PTEN + STK11, TSC2, PIK3R1, PIK3CA; AKT + mTOR, PIK3CA; mTOR + PTEN, AKT (Fig. 3).

### AEs

The most common treatment-emergent AEs, irrespective of attribution to study drug, are summarized by severity in Table 3 (severity graded per NCI CTCAE criteria v4.03). The most common grade 1–2 treatment-emergent AEs included nausea, vomiting, anorexia, diarrhea, fatigue, rash, hyperglycemia, and weight loss. Grade 3 to 5 treatment-related AEs included hyperglycemia (4/30; 13%), fatigue (2/30; 7%), hypertriglyceridemia (1/30; 3%), rash (2/20; 7%), diarrhea (2/30; 7%), creatinine increase (1/30; 3%), acidosis (1/30; 3%). Grade 5 acidosis was related to a hyperglycemia event. Patient was admitted with diabetic ketoacidosis (blood glucose >350 mg/dL). Hyperglycemia was likely induced by the mTOR inhibition in the setting of concomitant steroids. This event was attributed to the trial. After acute management of diabetic keto acidosis and resolution of hyperglycemia event patient was able to restart at reduced dose.

**TABLE 3:** AEs. Most common AEs are summarized by severity, severity based on NCI CTCAE criteria version 4.03

AE	G1	G2	G3	G4	G5	Total grade 1–2 (%)	Total grade 3–5(%)
Acidosis					1		1 (3)
ALT increased		1				1 (3)	0 (0)
Anemia		1				1 (3)	0 (0)
Anorexia	11	2				13 (43)	0 (0)
AST increased	1					1 (3)	0 (0)
Constipation	1					1 (3)	0 (0)
Creatinine increased		1	1			1 (3)	1(3)
Dehydration	1					1 (3)	0 (0)
Diarrhea	13	5	2			18 (60)	2(7)
Dizziness	2					2 (7)	0 (0)
Dry mouth	2					2 (7)	0 (0)
Dyspnea	1					1 (3)	0 (0)
Fatigue	2	4	2			6 (20)	2(7)
Headache	2					2 (7)	0 (0)
Hyperglycemia	1	5	4			6 (20)	4(13)
Hyperlipidemia	2					2 (7)	0 (0)
Hypertriglyceridemia	2		1			2 (7)	1 (3)
Hyperuricemia	1					1 (3)	0 (0)
Hypomagnesemia	1					1 (3)	0 (0)
Increased HbA1C	8					8 (27)	0 (0)
Mouth sore (mucositis)	7	1				8 (27)	0 (0)
Nausea	11	3				14 (47)	0 (0)
Pruritus	6	1				7 (23)	0 (0)
Rash	10	2	2			12 (40)	2(7)
Taste change	2					2 (7)	0 (0)
Tremor	1	1				1 (3)	0 (0)
Vomiting	11	1				12 (40)	0 (0)
Weight loss	7	2				9 (30)	0 (0)

Abbreviations: ALT, alanine aminotransferase; AST, aspartate aminotransferase.

## Discussion

This open-label single-institution phase I trial studied the safety and tolerability of the sapanisertib and metformin in combination with patients with advanced refractory cancer and AKT/mTOR/PI3K alterations. The safety profile of mTORC1/2 inhibitor sapanisertib in combination with metformin was generally tolerable, and toxicities observed were attributable to mTOR inhibition consistent with previously published literature. In addition, the combination of sapanisertib 4 mg plus metformin 1,000 mg was defined as the MTD and recommended phase II dose.

Previous groups have demonstrated treatment-related reductions in mTORC biomarkers, including TORC1/2 skin biomarkers (phosphorylated S6, 4EBP1, and PRAS40; ref. 28) which supports the dual TORC1/2 inhibitory activity of sapanisertib. In addition, a recently published phase I study of mTORC1/2 inhibitor sapanisertib (TAK-228) in advanced solid tumors, with an expansion in renal, endometrial, or bladder cancer confirmed the PD effect of sapanisertib on downstream effectors of TORC1 (p4EBP1 and pS6) and TORC2 (pPRAS40 and pNDRG1), with treatment-related decreases in p4EBP1, pS6, pPRAS40, and pNDRG1 using single-agent sapanisertib doses of ≥4 mg (17).

We demonstrate strong preliminary antitumor activity using sapanisertib in combination with metformin in a number of dose levels in molecularly selected patients of a range of tumor types: Disease control was observed in patients of various advanced malignancies harboring PTEN mutations and AKT/mTOR pathway alterations. Notably, 4 out of 24 evaluable patients achieved PR as best tumor response (17%) at a range of dose levels, emphasizing the antitumor activity of this combination. In addition to radiographic responses to this combination in this trial, several patients experienced a durable clinical benefit: out of all evaluable patient per RECIST, 79% (19 patients) had documented disease control (SD + PR). Three out of four PRs achieved were double mutants, that is, these patients had documented lesions in PTEN and STK11, PTEN and TSC2, AKT and mTOR, respectively, suggesting that perhaps hyperactivation of the pathway is important for responses to this combination. No objective responses were seen in patients harboring only *PIK3CA* mutations (*PIK3CA* E545K, *PIK3CA* H1047R, *PIK3CA* H1047L, other). Best responder was decrease of 15% in target lesions in a patient with liposarcoma harboring PIK3CA p.E545K with co-occuring NF1 p.S1420* alteration.

As P13K/mTOR pathway is concurrently activated in multiple tumors it may be worthwhile to explore combination therapy with agents with non-overlapping toxicity. In a translational study, it was shown that the double mutations hyperactivate PI3K signaling with increased tumor growth in preclinical models and also early clinical trial data showed that breast cancers harboring double mutations were more sensitive and responded better to PI3K inhibitors than those with a single mutation. It is possible that this “double mutation” in the same pathway is functional similarly in other tumors beyond breast cancer.

There are several limitations of this study, including the small number of patients treated, and the inclusion of many patients with comutations in addition to mTOR/AKT/PI3K pathway alterations, which may affect the underlying oncogenic driver pathway. Regardless, we show clinical activity in heavily pretreated patients which suggest that further study of sapanisertib in combination with metformin is warranted, particularly in patients with advanced malignancies harboring PTEN mutations and AKT/mTOR pathway alterations. This regimen could prove to be a highly effective treatment option, and a phase II study is warranted at the recommended phase II dose. In conclusion, sapanisertib had a manageable safety profile and the combination of sapanisertib and metformin was tolerable and exhibited preliminary therapeutic activity.

## Supplementary Material

Supplementary Figure 1, Supplementary Table S1, and Supplementary Table S2. Supplementary figure and tables

Representativeness of Study Participants Representativeness of Study Participants
