# Supplementary material for: Phase I Study of mTORC1/2 Inhibitor Sapanisertib (CB-228/TAK-228) in Combination with Metformin in Patients with mTOR/AKT/PI3K Pathway Alterations and Advanced Solid Malignancies
Source: Cancer Res Commun. 2024 Feb 12;4(2):378–87. doi: 10.1158/2767-9764.CRC-22-0260 (PMC10860536; doi:10.1158/2767-9764.CRC-22-0260)
Supplement: Supplementary Figure 1, Supplementary Table S1, and Supplementary Table S2. — Supplementary figure and tables [file crc-22-0260-s01.docx]

**Supplementary Figures and Tables:**

**Supplementary Figure 1. Survival plots.** Kaplan-Meier plot for (A) progression free survival and (B) overall survival for 30 patients treated on trial are shown. The median overall survival was 18 months (95% CI, 7.2-not estimated) and median progression-free survival was 6.0 months (95%CI, 2.8-14.6); median follow up was 19 months.

**Supplementary Table S1. Summary of dose-limiting toxicities in patients on study.**

| **Cohort** | **TAK-228 Dose** | **Metformin dose** | **Pts per cohort** | **DLTs** |
| --- | --- | --- | --- | --- |
| Cohort 1 | 3mg | 500mg | 3 | - |
| Cohort 2 | 3mg | 1000mg | 6 | 1 (G3 diarrhea) |
| Cohort 3 | 4mg | 1000mg | 10 | - |
| Cohort 4 | 4mg | 1500mg | 11 | 2 (G3 fatigue; G3 rash) |

**Supplementary Table S2. Partial responses achieved as best response, according to molecular profiling, in response-evaluable patients.**

| **Mutation** | **Tumor subtype** | **Best Response** | **Time to best response** | **Total treatment time** |
| --- | --- | --- | --- | --- |
| PTEN p.D252V; STK11 p.A318T | Breast | -88% | Cycle 18 | 24 cycles |
| PTEN p.W274 | Leiomyosarcoma | -35% | Cycle 6 | 20 cycles |
| TSC2p.F1510del; PTEN truncation exon 5 | Leiomyosarcoma | -33% | Cycle 12 | 15 cycles |
| AKT p.E17K; MTOR p.A1459D | Endometrial | -30% | Cycle 8 | Remains on treatment  (26 cycles) |

**clinicaltrials.gov NCT No: NCT03017833**
