# Supplementary material for: Phase I Study of mTORC1/2 Inhibitor Sapanisertib (CB-228/TAK-228) in Combination with Metformin in Patients with mTOR/AKT/PI3K Pathway Alterations and Advanced Solid Malignancies
Source: Cancer Res Commun. 2024 Feb 12;4(2):378–87. doi: 10.1158/2767-9764.CRC-22-0260 (PMC10860536; doi:10.1158/2767-9764.CRC-22-0260)
Supplement: Representativeness of Study Participants [file crc-22-0260-s02.docx]

| Representativeness of Study Participants | |
| --- | --- |
| Cancer type(s)/subtype(s)/stage(s)/condition | Advanced/Stage IV patients with cancer |
| Considerations related to: | |
| Sex | 63 % (N=19) female and 11 % male ( N=11) |
| Age | Median age at study enrollment, years (range) 57 (30–77) |
| Race/ethnicity | Asian 3 (10 %)  White 21 (70 %)  Hispanic 2 (7 %)  African-American 3 (10 %)  Other 1 (3 %) |
| Geography | As a referral clinic majority of the patients were from Texas. |
| Other considerations | This is a Phase 1 clinical trial in a large academic center. 30 patients with multiple advanced solid tumors were enrolled across 4 cohorts (3mg/500mg; 3mg/1000mg, 4mg/1000mg; 4mg/1500mg). 19 were female (63%), median age was 57 (range: 30–77), all were ECOG PS 1. Tumor types included sarcoma (6), breast (4), ovarian (4), head and neck (3), colorectal (2), lung (2), renal cell (2), endometrial (2), gastro-esophageal junction (1), prostate (1), stomach (1), urachus (1) and cervical cancer (1). |
| Overall representativeness of this study | The age distribution of our study is similar to the average age distribution of advanced solid tumor patients in Phase 1 clinical trials.  The male and female population and the race distribution is similar to that of an analysis of a 13,847 patients in 465 protocols Chihara D, et al Early drug development in solid tumours: analysis of National Cancer Institute-sponsored phase 1 trials. Lancet. 2022 Aug 13;400(10351):512-521. doi: 10.1016/S0140-6736(22)01390-3. PMID: 35964611; PMCID: PMC9477645. |
